# MRI insights into endometrial stromal tumors

**DOI:** 10.1186/s13244-026-02400-7

**Published:** 2026-09-21

**Authors:** Le Wang, Qi-Wen Hu, Yu Zou

**Affiliations:** Department of Radiology, Women’s Hospital School of Medicine Zhejiang University, Hangzhou, China

**Keywords:** Endometrial stromal tumors, Magnetic resonance imaging, Diagnosis, Differential

## Abstract

**Abstract:**

Preoperative misdiagnosis and the adoption of incorrect surgical approaches occur frequently in patients with endometrial stromal tumors (ESTs) because of their rarity, nonspecific clinical manifestations, and imaging features that highly overlap with those of other benign uterine tumors, thereby increasing the risk of secondary surgery and adverse outcomes. Thus, accurate diagnosis is essential, and following initial clinical and sonographic assessments, MRI serves as the cornerstone imaging technique for further characterization and differential diagnosis. Radiologists must be familiar with and adept at interpreting these images to narrow the differential diagnosis and at least suggest the possibility of EST. This article describes the imaging manifestations of various ESTs and highlights characteristic radiological features that enable radiologists to refine the differential diagnosis, thereby assisting clinicians in determining appropriate treatment strategies.

**Key Points:**

- **Question** Preoperative differentiation of ESTs from benign uterine lesions remains challenging and may result in the adoption of the incorrect surgical approach.
- **Findings** Key MRI features include homogeneous T2WI hyperintensity without speckles, T2WI hypointense bands, a worm‑like appearance, multilocular cystic changes, and feather‑like enhancement.
- **Critical Relevance Statement** This review comprehensively describes the MRI findings of ESTs and provides an educational summary based on tumor location, morphology, and key features to substantially improve diagnostic confidence.

**Graphical Abstract:**

**Figure:**
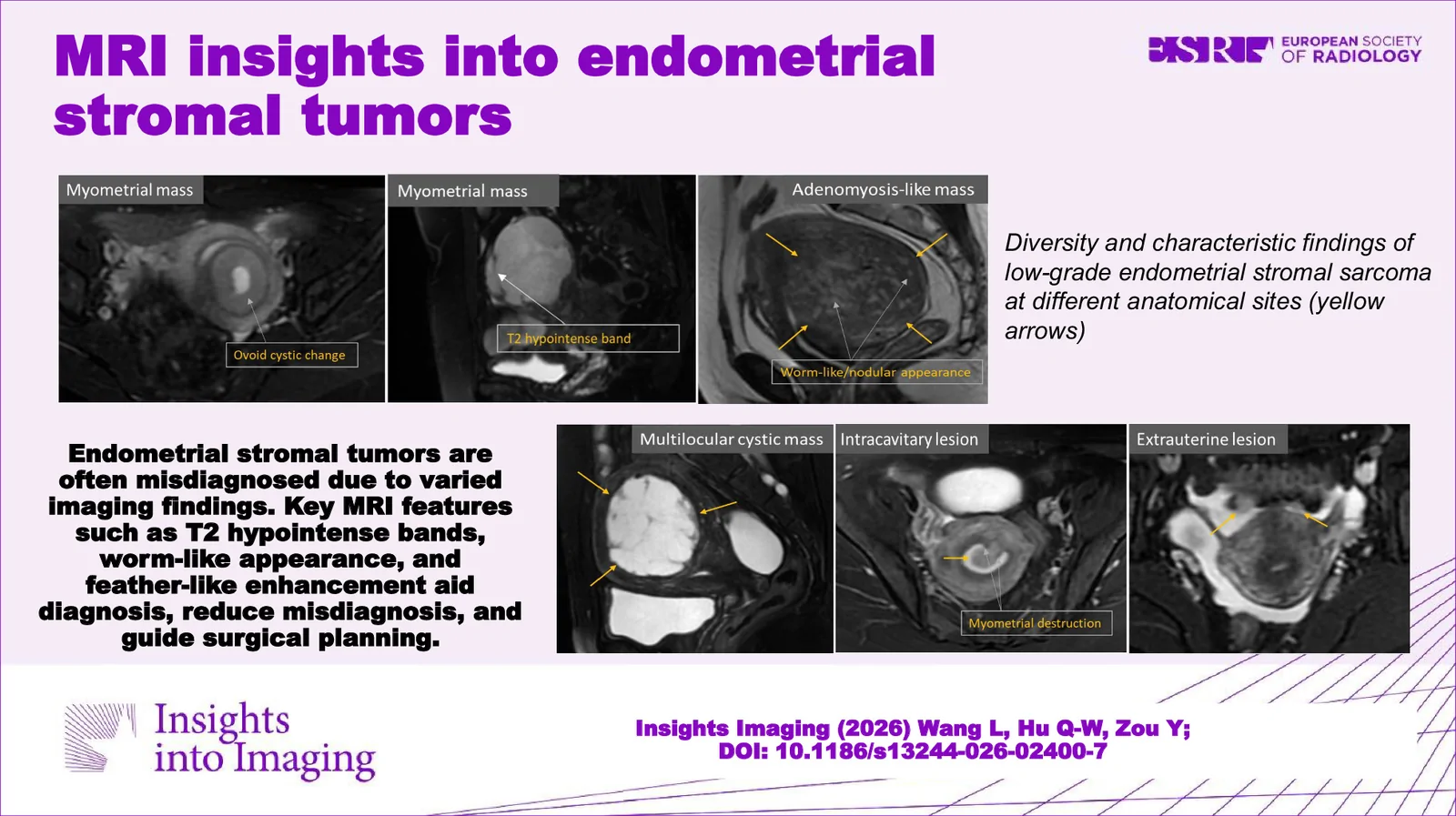

## Background

Endometrial stromal tumors (ESTs) are rare mesenchymal neoplasms, with an annual incidence of approximately 0.30 per 100,000 women [1]. The 2014 WHO classification categorized ESTs into four types on the basis of clinical and pathological features: endometrial stromal nodule (ESN), low-grade endometrial stromal sarcoma (LG-ESS), high-grade endometrial stromal sarcoma (HG-ESS), and undifferentiated uterine sarcoma (UUS) [2]. The current WHO (2020) classification lists UUS separately under “Endometrial Stromal and Related Tumors [3, 4]”. LG-ESS typically occurs in premenopausal patients (mean age 39 years), is characterized by slow growth, and has a favorable prognosis. In contrast, HG-ESS usually affects older patients (mean age of 61 years), shows aggressive growth, and has a poor prognosis [5, 6]. ESNs are benign tumors and, like ESS, occur primarily in perimenopausal and postmenopausal women [7]. Clinical presentations include vaginal bleeding, menorrhagia, abdominal pain, and a pelvic mass [8–10], with some cases being incidentally discovered during physical examination and lacking specificity.

ESTs can occur anywhere in the pelvis but are predominantly located within the uterine wall [11], and most involve the endometrium. Endometrial curettage may aid in preoperative diagnosis [12]. However, when the lesion is entirely intramural, curettage may not be helpful [13]. In such cases, imaging is crucial for diagnosing ESTs. Nevertheless, diagnostic imaging techniques, such as ultrasound, MRI, and positron emission tomography (PET), have not proven to be significantly helpful for the differential diagnosis of LG-ESS, which has a relatively high incidence [14–16], let alone for the rarer types. Owing to their diverse imaging manifestations, ESTs, both benign and malignant, are frequently misdiagnosed as benign uterine lesions, particularly leiomyomas, with the greatest diagnostic challenge observed in LG-ESS. Correctly differentiating malignant ESTs from benign uterine diseases is critical for guiding treatment and selecting the surgical approach. Uterine leiomyomas can be managed through various methods, including observation, hormonal therapy, uterine artery embolization, laparoscopic myomectomy, and simple hysterectomy [17]. In contrast, ESS requires staging surgery, typically involving total extrafascial hysterectomy with or without bilateral salpingo-oophorectomy, to minimize the risk of abdominal dissemination [18]. Some minimally invasive myomectomies (often involving morcellation) may inadvertently disseminate malignant cells throughout the abdomen, leading to iatrogenic metastatic disease with devastating consequences [19–21]. Therefore, summarizing the imaging characteristics of ESTs from existing imaging techniques is urgently needed. While not always enabling a definitive diagnosis, this can at least provide red flags to assist clinicians in adopting more appropriate treatment strategies.

Among the different imaging modalities, MRI has proven superior to ultrasound and computed tomography (CT) for characterizing indeterminate myometrial masses and is considered the preferred imaging method [22]. The existing studies on MRI for ESTs are primarily case reports. Despite extensive and ongoing research into more reliable diagnostic parameters, no effective diagnostic criteria exist to definitively determine the malignant sarcoma nature of a uterine mass before surgery. However, certain MRI findings may raise suspicion of malignancy and even suggest specific histological sarcoma subtypes [6]. For instance, some more general MRI features of uterine tumors should raise suspicion of malignancy (including irregular borders, intratumoral necrosis/hemorrhage, and low ADC values). Furthermore, specific characteristics may suggest particular histological subtypes, such as vascular/lymphovascular invasion associated with ESS, a “worm-like” appearance and intratumoral T2-weighted imaging (T2WI) hypointense bands in LG-ESS, and “feather-like” enhancement in HG-ESS [23–25]. Understanding the different histological EST subtypes, their specific MRI features, and their pathological basis allows for a more confident diagnosis and may indicate the correct histological subtype.

## MRI protocols

All MRI examinations were performed with the patient in the supine position using a pelvic phased-array coil on either a 1.5-T or 3.0-T scanner. The essential imaging protocol must include non-fat-suppressed T2WI in at least two orthogonal planes (axial and sagittal), as this sequence provides optimal delineation of uterine zonal anatomy and the tumor–myometrium interface. Fat-suppressed T2WI may be added as a complementary sequence to improve the conspicuity of typically T2WI hyperintense EST lesions, particularly when differentiating them from surrounding pelvic fat or in cases of extrauterine extension. Axial or sagittal T1-weighted images (T1-WI) (with or without fat suppression as needed) are needed for identifying intratumoral hemorrhage, necrosis, and cystic changes. Diffusion-weighted imaging (DWI) with a high b value (≥ 800 s/mm²) and corresponding apparent diffusion coefficient (ADC) maps is recommended for all suspected cases to assess true diffusion restriction. Finally, contrast-enhanced T1-WI (with optional fat suppression) should be obtained after intravenous administration of a gadolinium-based contrast agent (0.1–0.2 mmol/kg) to evaluate tumor enhancement patterns and detect vascular invasion. The detailed imaging parameters employed at our institution are provided in Table 1 for reference.

**Table 1:** Recommended magnetic resonance imaging protocol parameters for suspected ESTs

Parameters	T1-WI	T2-WI		Contrast enhanced		DWI
	Axial	Axial	Sagittal	Axial	Sagittal	Axial
Machine	GE Signa HDxt 1.5 T					
Sequence	LAVA	FSE	FSE	LAVA	LAVA	EPI-DWI
Repetition time (ms)	6.1	3600	3120	3.9	3.9	4600
Echo time (ms)	2.1	72	72	1.7	1.7	72.2
Field of view (mm^2^)	400 × 400	400 × 400	280 × 280	400 × 400	300 × 300	400 × 400
Slice thickness/gap (mm)	5.6/0	5.6/1	5.0/1	5.6/0	5.0/0	5.6/1
Machine	GE Signa Premier 3.0 T					
Sequence	LAVA	FSE	FSE	LAVA	LAVA	MUSE-DWI
Repetition time (ms)	4.5	6800	5020	3.9	5.0	3361
Echo time (ms)	1.2	80	68	1.8	2.3	58.8
Field of view (mm^2^)	320 × 320	320 × 320	280 × 280	320 × 320	280 × 280	340 × 340
Slice thickness/gap (mm)	1.6/0	4.0/1	4.0/1	1.6/0	2.0/0	4.0/1

*LAVA* liver acquisition with volume acceleration, *FSE* fast spin echo, *EPI-DWI* echo planar imaging diffusion weighted imaging, *MUSE-DWI* multiplexed sensitivity encoding diffusion weighted imaging

All T2-weighted images were acquired without fat suppression; fat-suppressed sequences may be added as needed

## ESN

### Clinical features

**Fig. 1:**
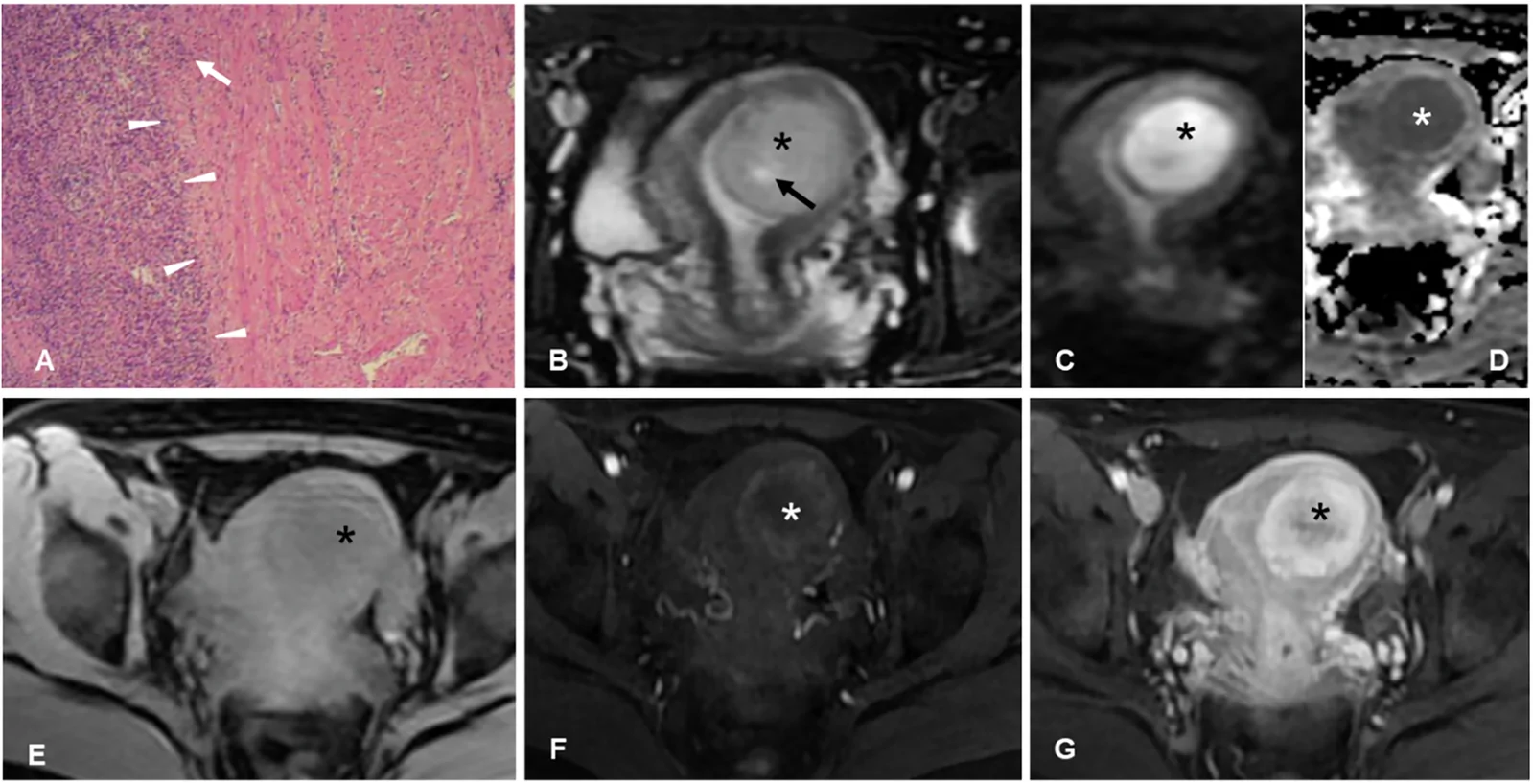
Pathological and magnetic resonance images of an ESN in a 42-year-old woman. Pathological image (**A**) demonstrating the ESN sharply demarcated from the surrounding myometrium. Axial T2WI (**B**) shows that the tumor (asterisk) has high T2WI signal intensity and an absence of a speckled appearance, along with small cystic changes within the tumor (arrow). DWI (**C**) and an ADC map (**D**) demonstrate restricted diffusion within the tumor. Axial T1-WI (**E**) depicts the tumor as slightly hypointense. Axial contrast-enhanced T1-WI (**F**, **G**) demonstrates marked progressive enhancement of the tumor

ESN is the least common type of EST and is predominantly found in perimenopausal women. It is a benign tumor composed of uniform, densely packed cells with oval nuclei, low mitotic activity, and scant cytoplasm, resembling the endometrial stromal cells of the proliferative phase of the menstrual cycle [26]. Microscopically, the lesion has a well-circumscribed border, occasionally showing finger-like protrusions into the myometrium; typically, there are ≤ 3 protrusions that are confined to within 3 mm of the edge [27] (Fig. 1). Lymphovascular space invasion (LVSI) is absent.

ESNs are mostly solid but may exhibit cystic changes. Several scholars suggest that ESNs frequently arise from the endometrium and are rarely found in the myometrium or subserosa [28]; however, a study encompassing 60 ESN cases reported 37 ESNs located entirely within the myometrium (intramural and subserosal) [7].

### MRI features

ESNs can present as mass-like or nodular lesions within the uterine wall or cavity. Given its rarity, the imaging literature on this entity is limited to case reports. Mass-like lesions are typically round or oval and well demarcated from the myometrium, which is consistent with the morphological features of benign or noninvasive tumors. On T2WI, the tumor often appears hyperintense, with a signal intensity similar to that of the normal endometrium [29], which is related to the tumor cells resembling proliferative-phase endometrial stromal cells. On DWI, ESN frequently exhibits marked hyperintensity with correspondingly low ADC values, reflecting the high cellular density of the tumor [30] (Fig. 1). However, these T2WI and DWI hyperintensities are not specific to ESN; LG-ESS can also exhibit similar features because the two tumors are cytologically highly similar, differing fundamentally only in the presence or absence of infiltration at the tumor–myometrium interface. Therefore, differentiation between them often requires postoperative pathology, and the specimen must include the tumor–myometrium interface [8, 31].

Conventional leiomyomas typically show low signal intensity on both T2WI and DWI, making differentiation from ESN relatively straightforward. However, some special subtypes of leiomyoma, such as cellular leiomyoma, can also appear hyperintense on T2WI and DWI because of their high cellular density. Unlike ESN, cellular leiomyomas often exhibit speckled or cord-like hypointense areas on T2WI and DWI within the mass, which correspond to collagen fibers [32–36] and result in a coarse signal appearance. This contrasts with the fine, homogeneous, and speckle-free signal of ESN (Table 2), which aids in diagnosis.

**Table 2:** MRI features of ESN, cellular leiomyoma, and conventional leiomyoma

ESN	Cellular leiomyoma	Conventional leiomyoma
- Regular morphology, appearing oval/round with well-defined borders. - Hyperintense on T2 and DWI. - Homogeneous and fine signal intensity on MRI without internal impurities.	- Regular morphology, appearing oval/round with well-defined borders. - Slightly hyperintense on T2 and DWI (with speckled or cord-like hypointense areas on T2WI within)	- Well-defined borders, with either regular or irregular shape - Hypointense on T2 and DWI (with cleft-like cystic T2 hyperintensity within)
		

**Fig. 2:**
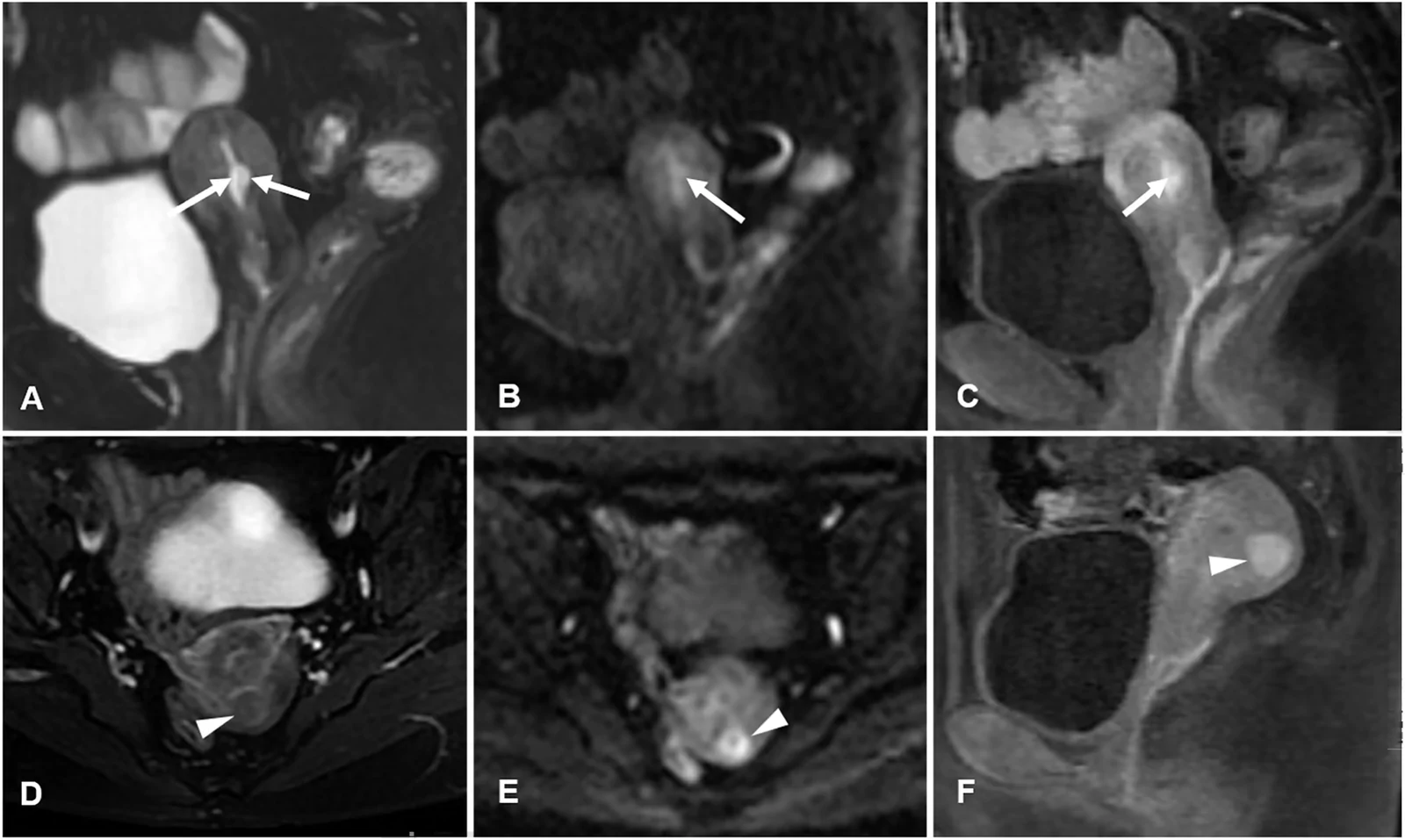
Magnetic resonance images of ESNs in a 63-year-old woman (**A**–**C**) and a 53-year-old woman (**D**–**F**). Sagittal T2WI (**A**) shows a lesion (arrows) located within the endometrial cavity, which has high signal intensity. Sagittal DW image (**B**) shows the lesion (arrow) with high signal intensity and ill-defined margins. Sagittal contrast-enhanced T1-WI (**C**) shows the lesion (arrow) with marked enhancement and well-defined margins. Axial T2W image (**D**) showing a lesion (arrowheads) located within the posterior myometrial wall, which is mildly hypointense (it is pathologically proven to be an ESN with smooth muscle differentiation). Axial DW image (**E**) showing a lesion (arrowheads) with high signal intensity. Sagittal contrast-enhanced T1-WI (**F**) showing the lesion (arrowheads) with marked enhancement and well-defined margins

When ESN presents as a small nodular lesion, its minute size makes imaging characterization difficult, and the lesion may be completely missed. An intracavitary ESN also requires differentiation from a polyp (Fig. 2). In imaging practice, when an endometrial nodule shows mildly hyperintense T2WI signals and markedly hyperintense DWI signals, the possibility of ESN should be considered.

## LG-ESS

### Clinical features

LG-ESS is the most common type of endometrial stromal tumor. It is a malignant tumor in which the cells resemble proliferative-phase endometrial stromal cells but demonstrate infiltrative growth, with or without lymphovascular invasion. It typically occurs in premenopausal women, is characterized by an indolent growth pattern, and has a relatively favorable prognosis (5-year survival rate of 91%) [5]. However, it is prone to venous invasion, and incomplete resection can lead to recurrence.

LG-ESS primarily arises within the myometrium or endometrium or involves both layers. Occasionally, it occurs in extrauterine sites such as the ovary, peritoneum, and vagina [37]. In contrast to ESN, LG-ESS has malignant growth characteristics, often accompanied by varying degrees of myometrial infiltration.

### MRI features

LG-ESS has diverse MRI findings, some of which are uncommon. On the basis of the predominant anatomical location, the tumor can be classified as having myometrium-predominant involvement, endometrial cavity involvement (with or without myometrial extension), or extrauterine involvement. The imaging features and key differential diagnoses for each location are described below to provide a practical diagnostic approach for clinical practice. Table 3 summarizes the main MRI findings and differential diagnoses for each category. Myometrium-predominant involvement in LG-ESS most commonly arises within the myometrium, where it can manifest with several distinct imaging features.

**Table 3:** MRI features of LG-ESS by predominant location

Predominant location	Imaging appearance	Key MRI features
Myometrium-predominant involvement	Mass-like	Well-defined solid mass; T2/DWI hyperintense (true restricted diffusion); T2 hypointense band; radial cystic change starting from the center
	Multilocular cystic	Well-defined multilocular cystic mass; cyst fluid similar to urine; fine T2-hypointense septa; DWI-hyperintense rim surrounding the cystic areas
	Adenomyosis-like	Ill-defined, infiltrative intramural lesion containing multiple nodular or “worm-like” T2/DWI hyperintensities
Endometrial cavity-predominant involvement	Intracavitary polypoid or irregular mass	T2/DWI hyperintense; marked enhancement; possible myometrial disruption (short band-like T2 hypointense structures angling toward the cavity)
Extrauterine involvement	Ovarian, bowel surface, or mesenteric nodules	Well-defined nodules; T2 and DWI hyperintense; marked enhancement; often “clean” pelvis

**Fig. 3:**
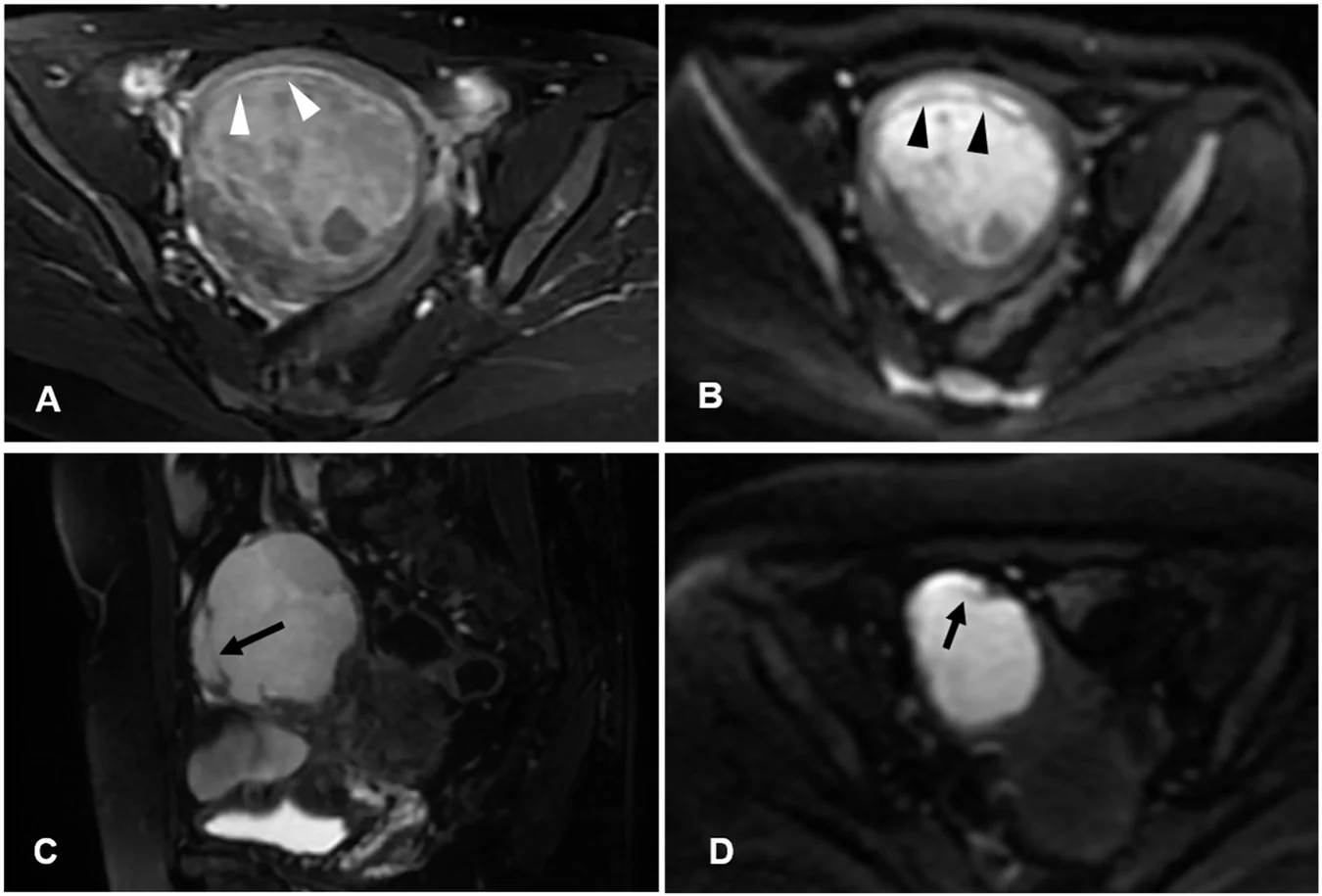
Magnetic resonance images of LG-ESS in a 52-year-old woman (**A**, **B**) and a 58-year-old woman (**C**, **D**). Axial T2W (**A**) and DW (**B**) images showing a well-defined, hyperintense tumor. Note the intratumoral T2WI hypointense band near the tumor margin (arrowheads). Sagittal T2W (**C**) and axial DW (**D**) images demonstrating an intratumoral T2WI hypointense band near the tumor margin (arrows)

**Fig. 4:**
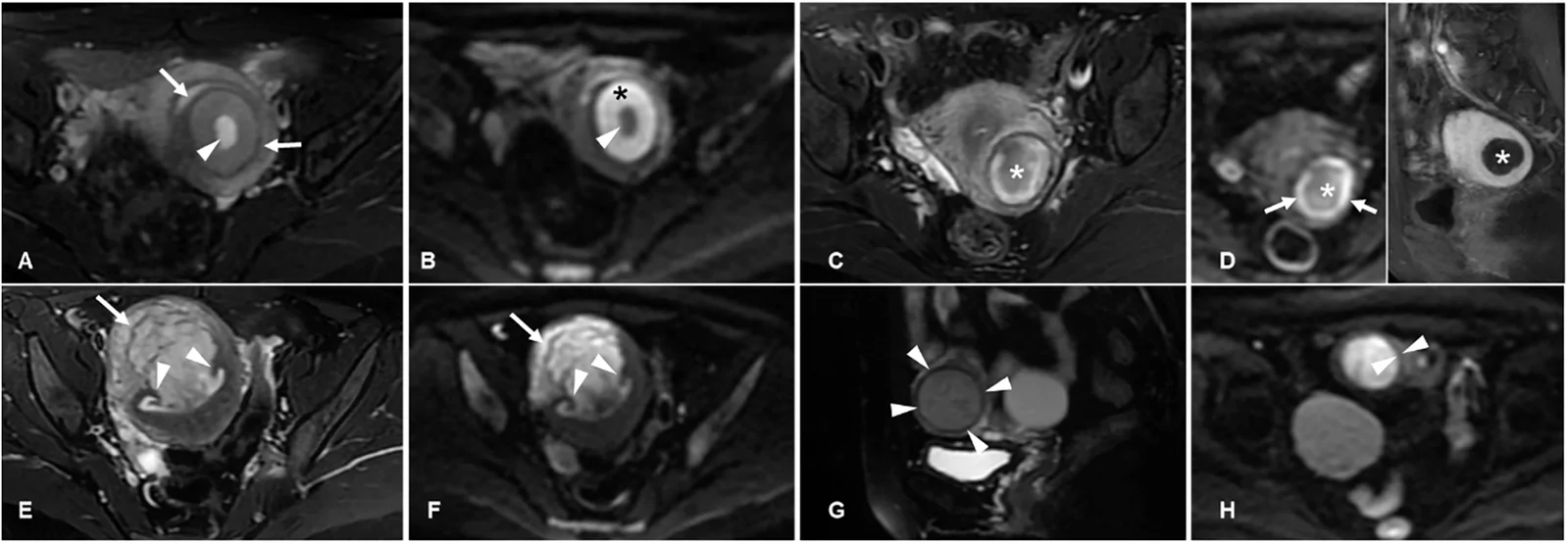
Pelvic magnetic resonance images of LG-ESS in four women: 33 years old (**A**, **B**), 46 years old (**C**, **D**), 46 years old (**E**, **F**), and 51 years old (**G**, **H**). Axial T2W (**A**) and DW (**B**) images show a well-defined T2WI hypointense rim (arrow) around the lesion, with a central oval cystic area (arrowheads) surrounded by DWI-hyperintense tumor tissue (asterisk). Axial T2W (**C**), axial DW, and sagittal contrast-enhanced T1-WI (**D**) reveal a well-defined posterior wall mass. A central round T2WI hypointense/DWI hypointense nonenhancing area (asterisk) indicates necrosis, encircled by a DWI-hyperintense tumor (arrows). Axial T2W (**E**) and DW (**F**) images show a mass in the anterior uterine wall eroding through the myometrium (arrowheads), with the disrupted myometrial bundles forming short, band-like T2WI/DWI hypointense structures angling toward the endometrial cavity. Multiple T2WI/DWI hypointense bands are also visible within the tumor (arrow). Sagittal T2WI (**G**) and axial DWI (**H**) depict a well-circumscribed round mass in the right uterine wall. A peripheral T2WI/DWI-hypointense rim (arrowheads) surrounds the lesion

Mass-like appearance—this is the most frequently reported presentation. It typically appears as a round or oval solid mass located within the myometrium, with regular morphology and well-defined borders. The mass usually shows hyperintensity on T2WI, a high signal on DWI, and low ADC values. In addition to having imaging features similar to those of ESN, LG-ESS often infiltrates the surrounding myometrium, forming a characteristic intratumoral T2WI hypointense band. Histologically, this corresponds to preserved normal myometrial bundles separated by clusters of infiltrating tumor cells [24, 38] (Fig. 3). A peripheral T2WI hypointense rim may also form around the tumor (Fig. 4), which likely reflects a layer of fibrous tissue and/or reduced free water resulting from myometrial deformation due to tumor expansion [39]. However, the specificity of this rim sign is controversial, as it can also be observed in other myometrial tumors [40]. Some degenerated, or special subtypes of leiomyomas may also show T2WI or DWI hyperintensity due to degeneration, edema, or high cellularity, leading to substantial imaging overlap with LG-ESS [41]. Beyond the fine, homogeneous, and speckle-free T2WI signal (differentiation of uterine LG-ESS from rare leiomyoma variants by magnetic resonance imaging (MRI)), the infiltrative MRI features of ESS—such as the intratumoral T2WI hypointense band—are key to differentiation. In addition, several less frequently reported signs deserve attention (Fig. 4): (1) the incidence of cystic degeneration or necrosis in ESS is greater than that in rare leiomyoma variants [42]. In contrast to the cleft‑like cystic degeneration or necrosis commonly seen in leiomyomas, cystic degeneration in ESS tends to spread radially from the center to the periphery of the mass [11] and typically presents as round or oval areas with sharp margins. In some cases, a solitary round cystic area is seen within the lesion, sharply demarcating it from the surrounding solid tumor components, which may be of some indicative value. (2) When a myometrial tumor invades the inner myometrium, MRI may reveal a pair of disrupted free ends of myometrial bundles presenting as short, band-like T2WI hypointense structures curving toward the endometrial cavity. This finding is a noteworthy imaging feature that has not been specifically highlighted in previous reports. Unlike intramural LG-ESS, uterine leiomyosarcoma typically shows irregular morphology, indistinct borders, an intermediate signal on T2WI, marked enhancement, and a high propensity for extensive intratumoral necrosis and hemorrhage [43], often presenting with characteristic central necrotic areas and fluid‒fluid levels due to hemorrhage [23]—features that typically aid in differentiation, although occasional cases may remain challenging to diagnose. Notably, the abovementioned infiltrative features of LG-ESS are usually not isolated but often coexist.

**Fig. 5:**
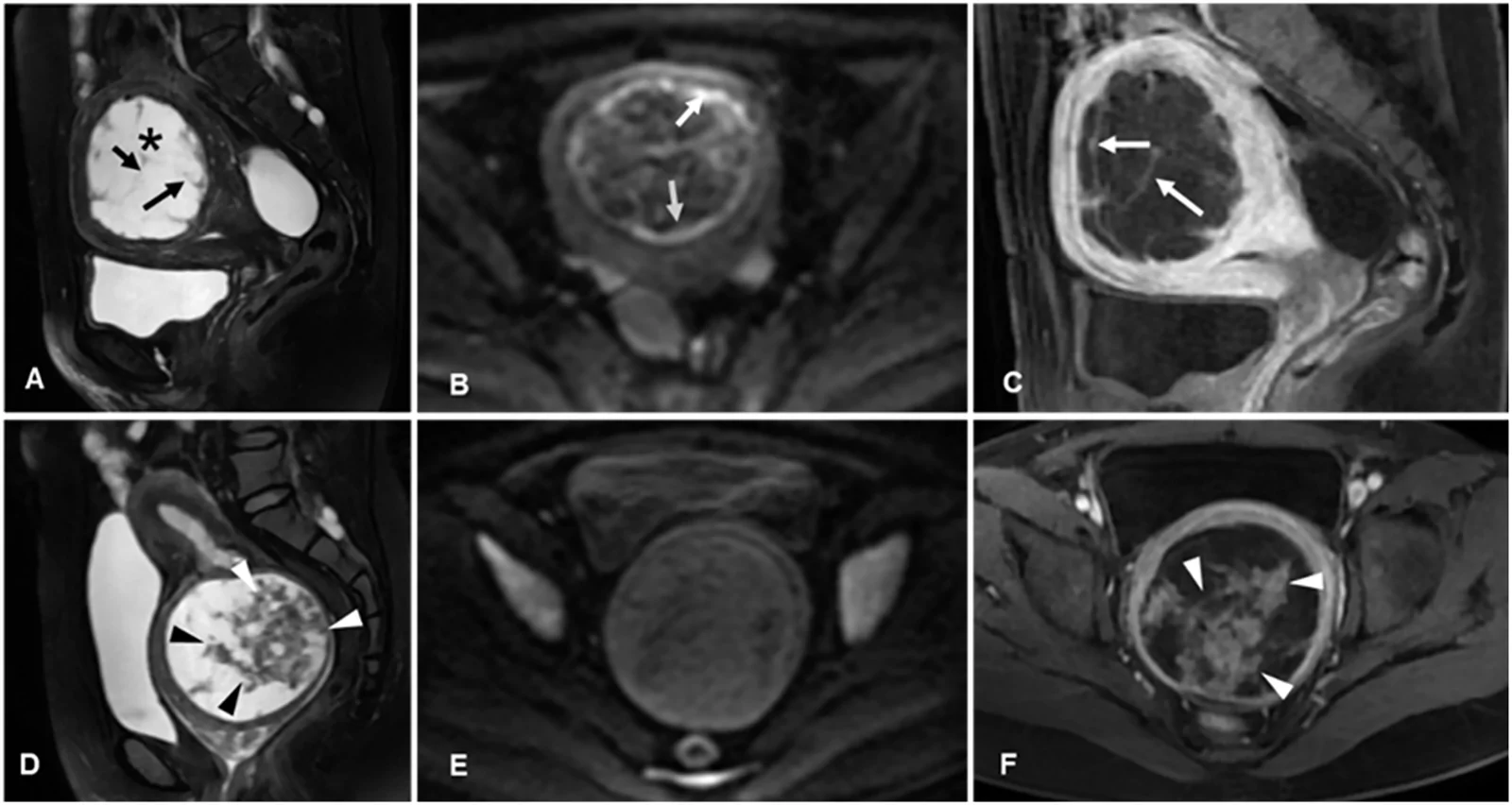
**A**–**C** Low-grade LG-ESS in a 53-year-old woman. **D**–**F** Degenerated leiomyoma with cystic changes in a 46-year-old woman. Sagittal T2W image (**A**) showing a well-defined cystic mass within the myometrium (asterisk), which is hyperintense on T2WI with multiple thin T2WI hypointense “septa” (arrows). Axial DW image (**B**) showing cord-like hyperintense signals surrounding the lesion (arrows). Contrast-enhanced image (**C**) demonstrating marked enhancement of the “septa”. Sagittal T2W image (**D**) displaying a well-defined cystic-solid mass protruding into the cervical canal from the submucosa, predominantly cystic with patchy and cord-like T2WI hypointense areas (arrowheads), which demonstrate marked enhancement on the image (**F**). Axial DW image (**E**) demonstrates no hyperintensity within the lesion

Multicystic appearance—in rare instances, LG-ESS may present as a well-defined, multicystic mass located within the myometrium, with multiple fine hypointense septa and a cyst fluid signal intensity nearly identical to that of urine on T2WI (Fig. 5). The pathogenesis of this multicystic appearance remains inconclusive. Several studies have suggested an association with cystic dilatation of the endometrioid glands [44, 45]. In contrast, another report revealed no evidence of endometrial glandular components in cystic lesions but revealed endometrial stromal differentiation cells surrounding the cystic degenerative areas [46]. Notably, the latter finding—stromal differentiation cells surrounding cystic areas—corresponds to the DWI-hyperintense rim around the cystic area in Fig. 5B, indicating the presence of tumor components. Although the exact mechanism underlying this multicystic appearance of LG-ESS remains ambiguous, a well-defined intramural multicystic mass is considered a characteristic feature of LG-ESS and can greatly increase diagnostic confidence.

**Fig. 6:**
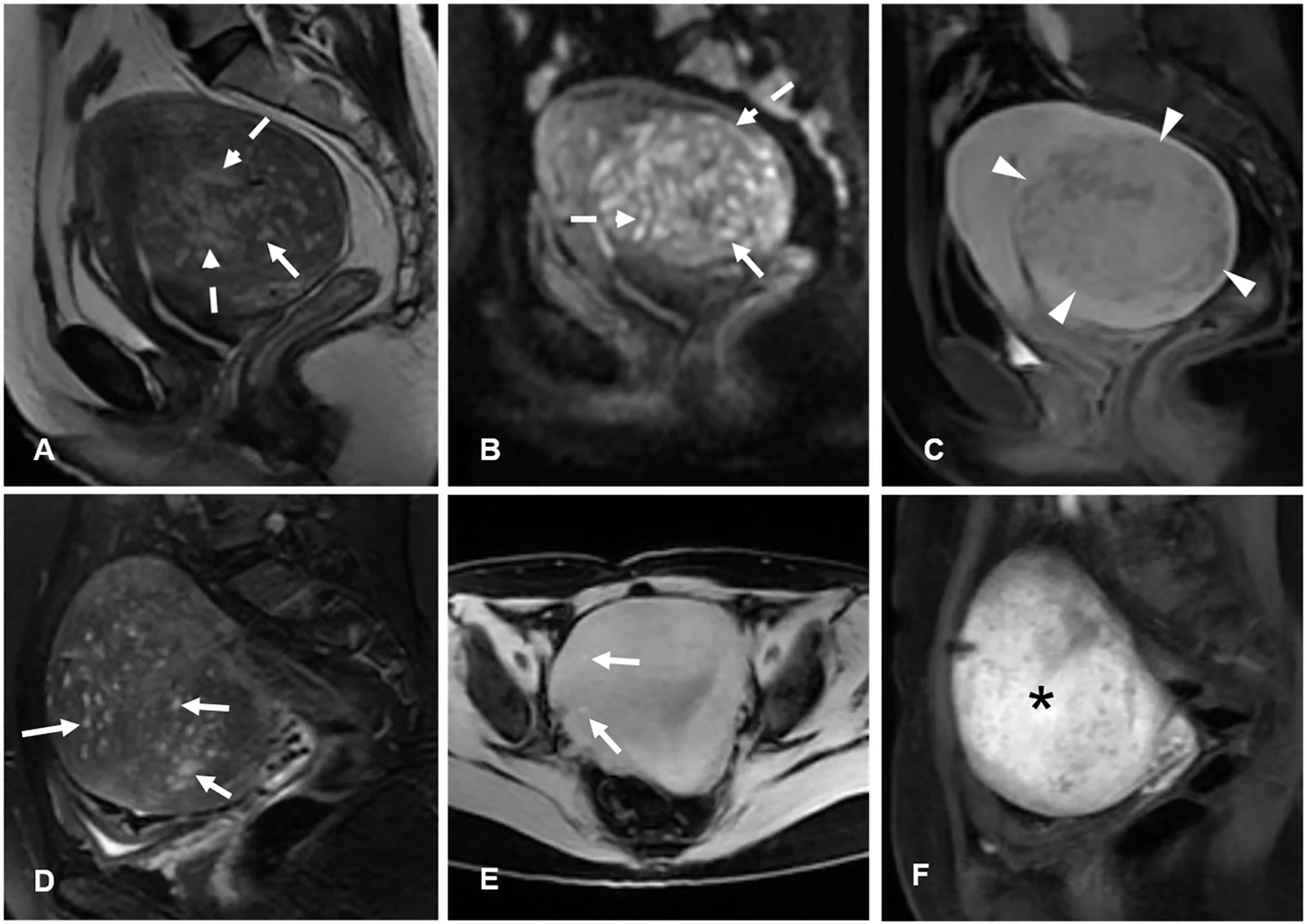
Magnetic resonance images of LG-ESS (**A**–**C**) and adenomyosis (**D**–**F**) in a 50-year-old woman. Sagittal T2W (**A**) and DW (**B**) images demonstrate a mass in the posterior uterine wall with ill-defined margins. Within the mass, “worm-like” (dotted arrows) and nodular (arrows) areas of high signal intensity are visible on both T2WI and DWI. Sagittal contrast-enhanced T1-WI (**C**) shows relatively clear delineation of the tumor margins and enhancement slightly lower than that of the surrounding myometrium. Sagittal T2W image (**D**) shows uterine enlargement with a thickened uterine wall containing multiple nodular T2WI hyperintense foci. Axial T1-WI (**E**) reveals punctate high-signal foci within the lesions. Sagittal contrast-enhanced T1-WI (**F**) shows lesion enhancement similar to that of the myometrium, without clear boundaries

Adenomyosis-like appearance—this pattern presents as a poorly defined mass within the uterine wall, containing multiple “worm-like,” nodular hyperintensities on T2WI and DWI (Fig. 6). The peripheral nodules represent myometrial infiltration; when the lesion invades intramural veins, it fills and distends these myometrial veins, manifesting as a characteristic worm-like appearance [47]. This LG-ESS variant is often confused with adenomyosis. Adenomyosis typically presents as thickened myometrium with T1 hyperintense foci, which are crucial for differentiation, and multiple punctate T2WI hyperintense foci within the thickened hypointense myometrial wall on T2-WI (T2-weighted images) (Fig. 6), but with a low DWI signal and myometrium-like enhancement. In contrast, adenomyotic LG-ESS displays characteristic worm-like patterns that are visible across planes, distinguishing it from adenomyotic cysts/nodules.

This rare variant of LG-ESS requires differentiation from leiomyomas with hyaline or hydropic degeneration. Features that aid differentiation include well-defined borders, relatively homogeneous T2 hyperintensity, and fine, regular internal septations in LG-ESS. In contrast, degeneration in leiomyomas typically begins in the periphery and progresses centrally, leaving behind irregular solid leiomyomatous components (Fig. 5).

## Endometrial cavity-predominant involvement

This pattern of LG-ESS presents as a mass predominantly or completely located within the endometrial cavity, which may appear polypoid, lobulated, or irregular. On MRI, the mass shows hyperintensity on T2WI and DWI, with marked contrast enhancement due to its hypervascular nature [48, 49]. The differential diagnosis includes endometrial polyp, submucosal leiomyoma, and endometrial carcinoma.

**Fig. 7:**
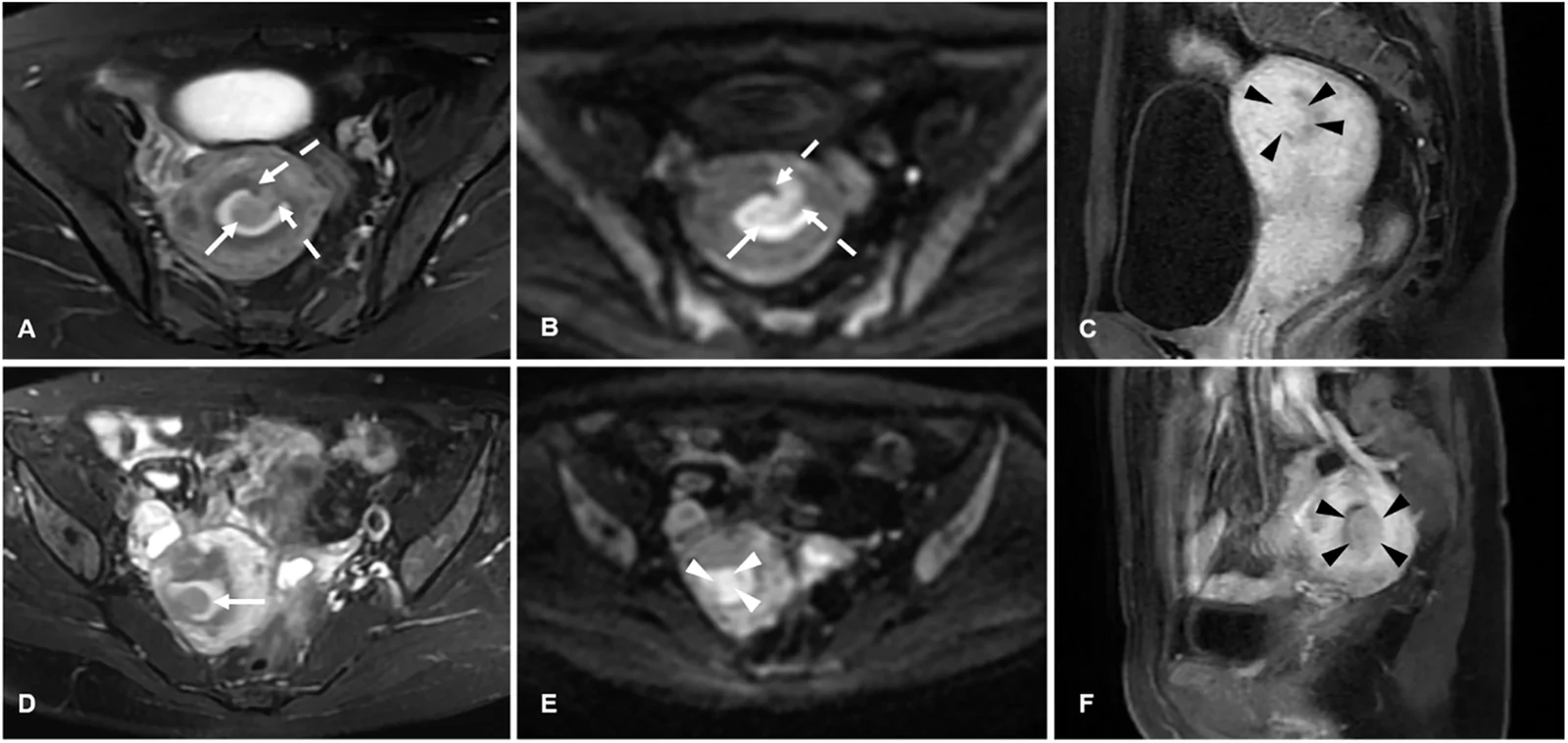
LG-ESS and pelvic magnetic resonance images from a 46-year-old (**A**–**C**) woman and a 54-year-old (**D**–**F**) woman. Axial T2W (**A**) and DW (**B**) images show a small nodular lesion protruding into the uterine cavity, appearing hyperintense on T2WI (relative to the uterine junctional zone) and hyperintense on DWI (arrows). Disruption of the adjacent junctional zone can be seen (dotted arrows). Sagittal T1-WI-enhanced scan (**C**) shows marked enhancement of the lesion (arrowheads). Axial T2W image (**D**) showing a T2WI mildly hyperintense nodule protruding into the uterine cavity (arrows), and axial DW image (**E**) showings lesion with a hyperintense signal (arrowheads) but with indistinct borders (consistent with the signal of the surrounding endometrium); a sagittal T1-WI-enhanced (**F**) image shows that the degree of lesion enhancement is slightly lower than that of the surrounding myometrium (arrowheads)

A well-circumscribed lesion (Fig. 7) requires differentiation from endometrial polyps and submucosal leiomyomas, which typically show hypointense signals on T2WI and DWI, making differentiation relatively straightforward. However, some degenerated submucosal leiomyomas may also appear hyperintense on T2WI and DWI, limiting the reliability of signal intensity alone. When the tumor invades the underlying myometrium, MRI may reveal disrupted myometrial bundles presenting as short, band-like hypointense structures curving toward the endometrial cavity on T2WI. This finding may be of some indicative value, but its diagnostic significance requires further validation.

**Fig. 8:**
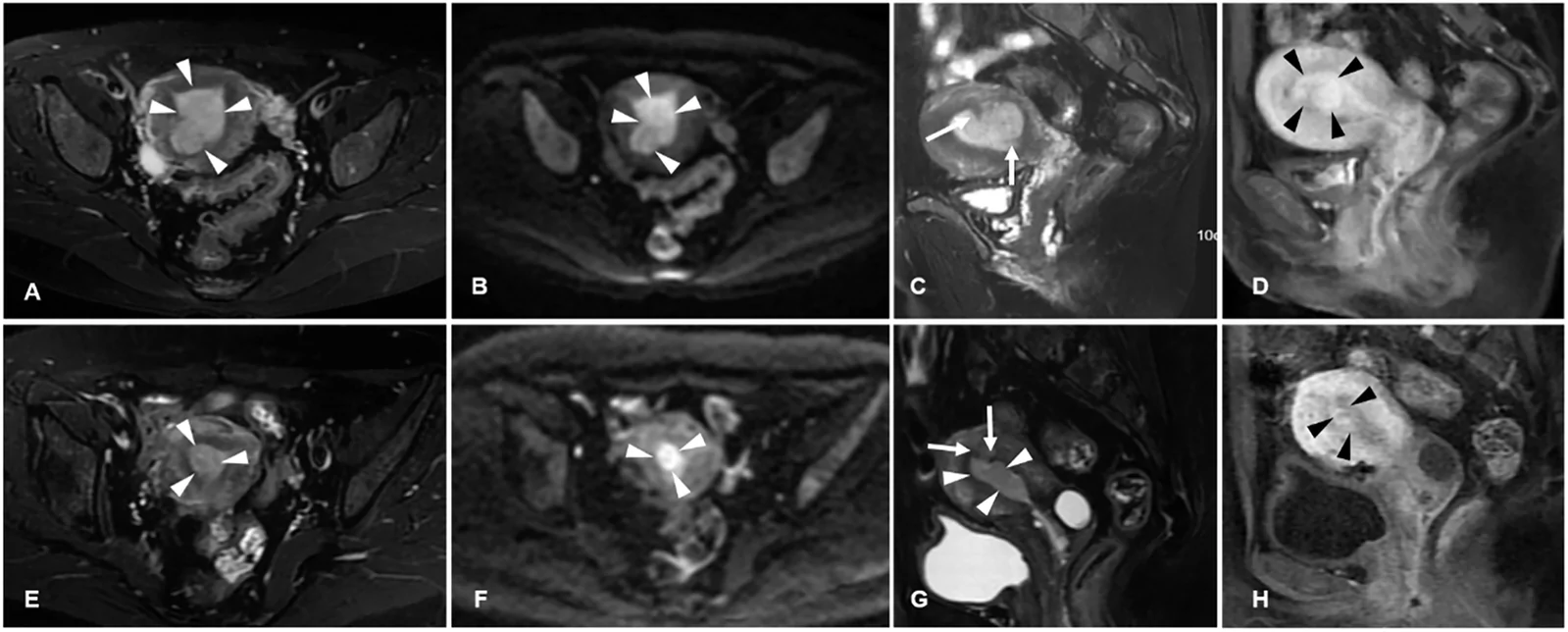
LG-ESS from pelvic magnetic resonance images of a 50-year-old (**A**–**D**) woman and a 55-year-old (**E**–**H**) woman. Axial T2W (**A**) and DW (**B**) images showing a poorly defined mass extending from the endometrial cavity into the myometrium, predominantly located within the cavity, appearing hyperintense on T2WI and DWI (arrowheads). Sagittal T2W image (**C**) showing disrupted myometrial bundles angling toward the endometrial cavity (arrows). Sagittal T1-weighted contrast-enhanced image (**D**) showing marked enhancement of the lesion (arrowheads). Axial T2W (**E**) and DW (**F**) images showing an irregular mass within the endometrial cavity, which appears hyperintense (arrowheads). Sagittal T2-WI (**G**) showing disrupted myometrial bundles angling toward the endometrial cavity (arrows). Sagittal T1-weighted contrast-enhanced image (**H**) showing marked enhancement of the lesion (arrowheads)

An irregular lesion (Fig. 8) requires differentiation from endometrial carcinoma. Compared with endometrial carcinoma, LG-ESS typically shows more prominent enhancement and a greater likelihood of the above-described myometrial disruption. In contrast, endometrial carcinoma usually increases only mildly and tends to invade the myometrium in an infiltrative manner.

**Fig. 9:**
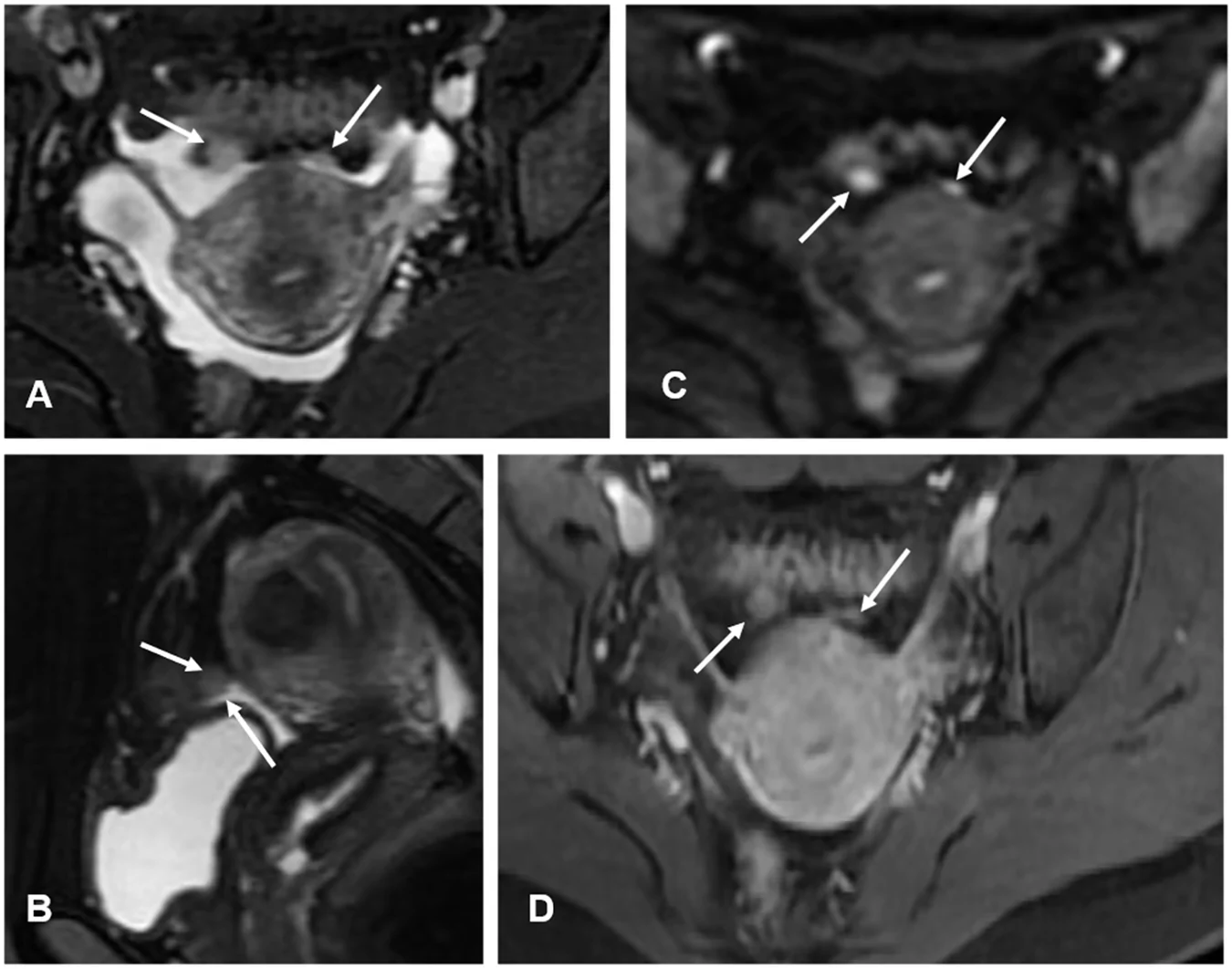
**A**–**D** LG-ESS in a 34-year-old woman. Axial (**A**) and sagittal (**B**) T2W images showing hyperintense nodules on the serosal surfaces of the bowel and the anterior uterine wall (arrows). Axial DWI image (**C**) demonstrating markedly hyperintense lesions. Axial T1-weighted contrast-enhanced image (**D**) showing significant enhancement of the lesions

Extrauterine involvement is rare in LG-ESS, and most commonly in the ovary because of malignant transformation of the ectopic endometrium [50]. Many cases result from pelvic dissemination/metastasis following prior misdiagnosis of a benign uterine “fibroid” and morcellation [13]. Typical primary extrauterine LG-ESS originating from endometriosis presents as well-defined nodules with mildly T2 hyperintense signals attached to the uterine serosa, bowel surface, or ovarian surface, showing marked hyperintensity and significant enhancement on DWI (Fig. 9). Notably, these lesions often have sharp borders, without significant ascites or pelvic sidewall peritoneal thickening, making the pelvis appear relatively “clean”. These signs help differentiate these tumors from other pelvic tumors, such as primary peritoneal carcinoma or ovarian cancer metastases.

**Fig. 10:**
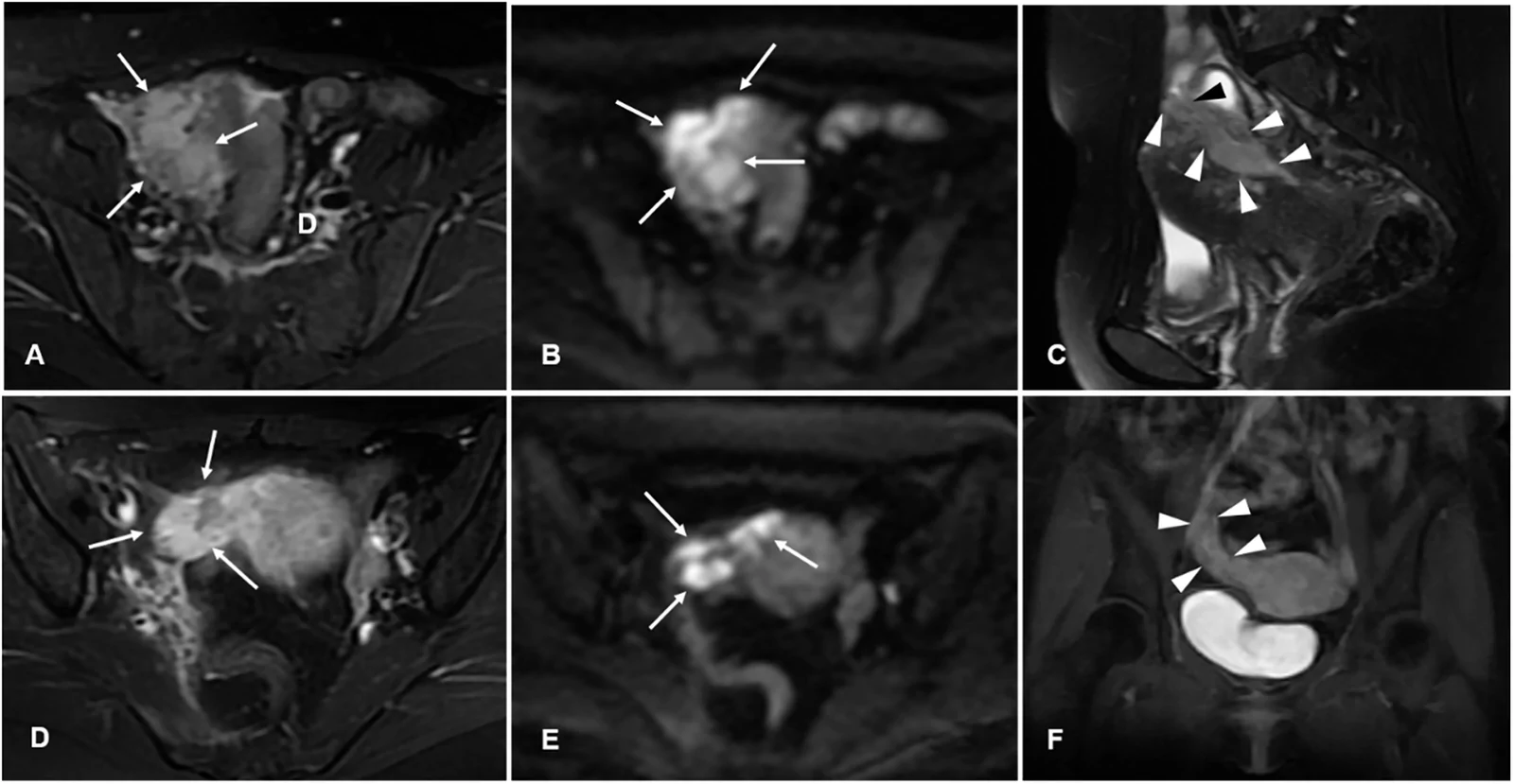
LG-ESS from pelvic MRI of a 43-year-old (**A**–**C**) woman and a 53-year-old (**D**–**F**) woman. Axial T2WI (**A**) and DWI (**B**) images showing ill-defined hyperintense lesions in the right posterior uterine wall and parametrium (arrows). Sagittal T2W image (**C**) revealing tumor extension beyond the uterus involving the right ovary and parametrial vessels (arrowheads). Axial T2W (**D**) and DW (**E**) images demonstrating ill-defined hyperintense lesions predominantly in the right parametrium with uterine wall involvement (arrows). Coronal T1-weighted contrast-enhanced image (**F**) displaying tumor extension along the parametrial veins

Another interesting feature that could potentially differentiate LG-ESS from other tumors is the presence of extrauterine masses within periuterine vessels or adjacent to the uterosacral ligaments/fallopian tubes (Fig. 10). This specific feature of intravascular extension is characteristic of this sarcoma subtype and explains its former term “endolymphatic stromal myosis”. This appearance should be differentiated from intravenous leiomyomatosis, which is a rare disseminated form of benign leiomyoma with similar imaging features but lacks the aggressive characteristics of ESS, such as irregular borders and intratumoral necrosis.

## HG-ESS

### Clinical features

HG-ESS is a highly malignant endometrial stromal tumor characterized microscopically by uniform high-grade round and/or spindle cells, sometimes with a low-grade component. HG-ESS primarily affects older women, who are often perimenopausal or postmenopausal. It is more aggressive, grows rapidly, has extensive local invasion, and has a poor prognosis (5-year survival rate of only 33%) [5, 39]. Compared with LG-ESS, HG-ESS often presents at an advanced stage and is associated with frequent recurrences [51]. Morphologically, they exhibit either destructive or permeative (infiltrative) growth patterns. In approximately half of the tumors, a low-grade component with a fibroblastic/myxoid or less common typical morphology can be found, presenting a biphasic appearance, and these low- and high-grade tumor areas may not be sharply demarcated from each other [52]. This makes differentiating high- and low-grade ESS challenging.

### MRI features

**Fig. 11:**
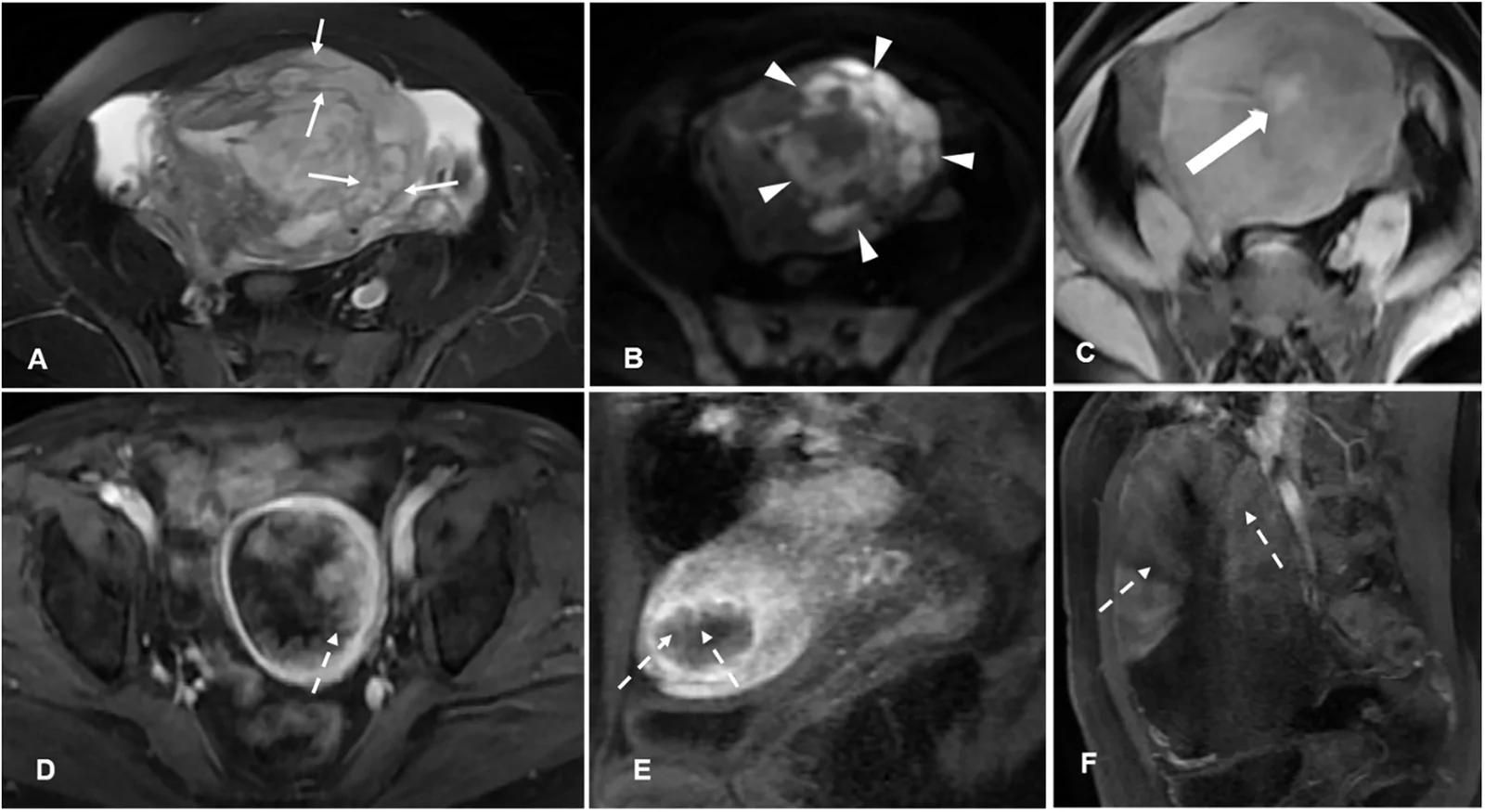
HG-ESS from pelvic MRI of a 49-year-old (**A**–**C**), 69-year-old (**D**), 39-year-old (**E**), and 37-year-old (**F**) woman. Axial T2W (**A**) and DW (**B**) images showing ill-defined hyperintense lesions within the uterus (arrowheads), containing multiple thin septations and demonstrating a worm-like appearance (arrows). Axial T1-WI (**C**) revealing hemorrhagic foci within the lesion (broad arrow). Axial (**D**) and sagittal (**E**, **F**) T1-weighted contrast-enhanced images displaying characteristic “feather-like” enhancement of the lesions (dotted arrows)

There is significant overlap in MRI features between HG-ESS and LG-ESS [53]. HG-ESS also tends to present as large, heterogeneous, mildly hyperintense masses with intratumoral T2hypointense bands and worm-like appearances. on T2WI. Differentiation is particularly difficult when the tumor is small and shows minimal surrounding tissue destruction/invasion. However, some findings are more suggestive of its higher-grade nature and point towards the correct diagnosis. These features include larger areas of hemorrhage and necrosis, prominent lymphovascular invasion, and “feather-like” enhancement after intravenous contrast administration (Fig. 11). Studies suggest that feather-like enhancement achieves up to 95% diagnostic accuracy [23, 24, 54]. Feather-like enhancement was used to describe fine, wispy enhancement interspersed within tumors, likely indicating the more destructive myometrial infiltration characteristic of HG-ESS, which is distinct from the finger-like protrusions of LG-ESS [37]. On T2-weighted sequences, the preserved hypointense myometrial fiber bundles are typically thinner, more scattered, and harder to delineate than they are in low-grade disease because of greater myometrial destruction. Notably, the imaging features of HG-ESS are derived from a limited number of case series, reflecting the rarity of this tumor.

## Treatment and follow-up

ESN has an excellent prognosis, and patients can be cured by hysterectomy [7]. Conservative treatment with mass excision is only possible if margins can be fully assessed, which occurs in very rare circumstances [55].

**Fig. 12:**
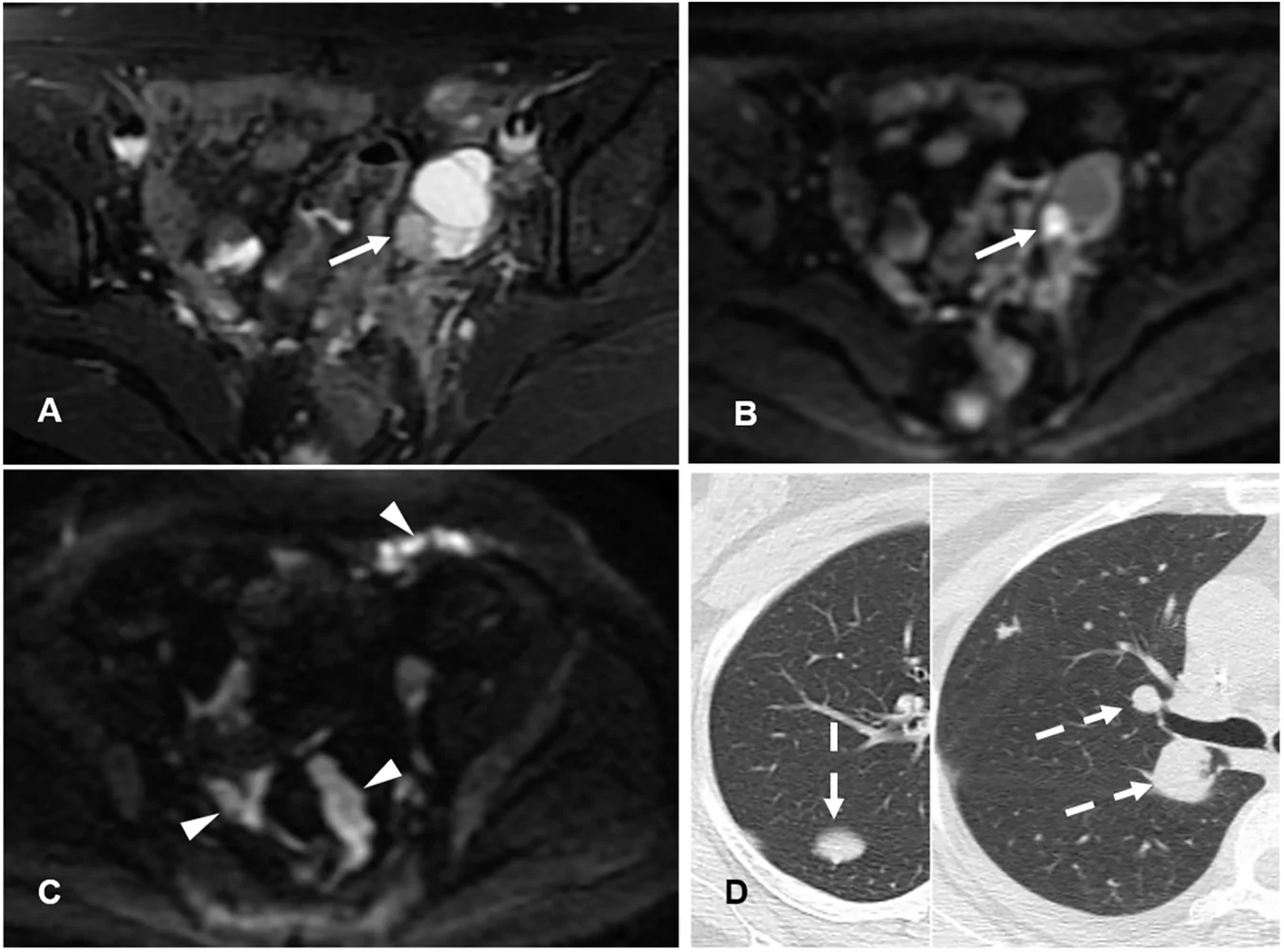
LG-ESS from pelvic MRI and chest CT of a 35-year-old (**A**, **B**) woman and a 46-year-old (**C**, **D**) woman. Axial T2W (**A**) and DW (**B**) images showing an absent uterus with a hyperintense nodular recurrent lesion on the left ovarian surface (arrows). Axial DW (**C**) image demonstrating multiple hyperintense metastatic lesions in the mesentery and anterior abdominal wall (arrowheads). Chest CT image (**D**) revealing well-defined nodules pathologically confirmed as metastases (dotted arrows)

Surgery is the primary treatment for both high-grade and low-grade ESS confined to the uterus [56]. The main surgical procedure is hysterectomy with bilateral salpingo-oophorectomy. Studies indicate a higher recurrence risk in patients with ovarian preservation [57, 58]. Tumors can recur in the ovaries, disseminate within the abdomen, or metastasize distantly (e.g., to the lungs) (Fig. 12). Current guidelines [59] acknowledge the limited role of adjuvant radiotherapy in reducing locoregional recurrence, particularly in patients with stage II+ disease. Analyses of large databases (SEER) indicate that while radiotherapy may improve local control, this does not translate into an overall survival benefit, and evidence from randomized trials is lacking. Given its defined side-effect profile and absence of a survival advantage, adjuvant radiotherapy is not routinely recommended.

The 5-year survival rate for patients with FIGO stage I ESS ranges from 54% to nearly 100%, and for patients with stage II ESS, it is approximately 30%. Survival for patients with advanced stages (III and IV) is only approximately 11%. Owing to the tendency for late recurrence, long-term regular follow-up is essential, with a recommended frequency of every 3 months during the first year post-operatively, every 6 months from years 2 to 5, and annually thereafter [60]. The cornerstone of each visit lies in a detailed medical history and a pelvic/abdominal physical examination. With respect to imaging surveillance, an indication-driven and stratified approach should be followed. Owing to its safety, pelvic ultrasound can serve as a routine monitoring tool; when ultrasound findings are indeterminate or clinical suspicion of pelvic recurrence arises, pelvic MRI is recommended. For the chest evaluation, chest X-ray or CT should only be indicated when clinical symptoms or signs suggestive of pulmonary metastasis are present. During long-term follow-up (e.g., after 5 years), chest X-ray may be prioritized to reduce radiation exposure. In cases of high recurrence risk or strong clinical suspicion of widespread metastasis, CT of the chest, abdomen, and pelvis may be considered, supplemented by PET-CT if necessary [61]. Currently, tumor markers or routine blood tests are not recommended for routine surveillance in asymptomatic patients. Given concerns regarding radiation exposure, frequent routine asymptomatic imaging surveillance after primary treatment is not advised [62]. In addition to exposure to exogenous estrogen—a negative prognostic factor driven by high estrogen receptor (ER) expression in ESS [63]—a favorable prognosis depends on tumor stage, depth of myometrial invasion, adjuvant therapy, and performance of bilateral salpingo-oophorectomy [64].

## Conclusion

In summary, MRI is the preferred imaging modality for the detection and diagnosis of ESS. Radiologists should be familiar with the MRI manifestations of ESTs and meticulously evaluate the myometrial interface at the tumor margins. Although there are currently no effective screening programs or international guidelines for reliably diagnosing uterine sarcoma preoperatively, several characteristic imaging findings strongly suggest ESS. In daily practice, high suspicion for EST should arise when a postmenopausal woman not on hormone replacement therapy presents with vaginal bleeding and a uterine nodule (presumed fibroid) exhibiting the characteristic EST features described above. In such cases, further radiological evaluation may aid in reaching the correct diagnosis to guide appropriate surgical management and improve survival rates. Future prospective studies on ESTs that integrate multiparametric quantitative analyses such as DWI, ADC, and dynamic contrast-enhanced MRI kinetic curves hold promise for establishing a more accurate noninvasive preoperative diagnostic model.

## Data Availability

The data of cases in the manuscript are available from the corresponding author upon reasonable request.

## Abbreviations

ADC: Apparent diffusion coefficient
CT: Computed tomography
DWI: Diffusion-weighted imaging
ESN: Endometrial stromal nodule
ESS: Endometrial stromal sarcoma
EST: Endometrial stromal tumors
HG-ESS: High-grade endometrial stromal sarcoma
LG-ESS: Low-grade endometrial stromal sarcoma
MRI: Magnetic resonance imaging
PET: Positron emission tomography
T1-, T2-WI: T1-, T2-weighted images
